# Global burden of pain in malignant bone tumors: a meta-analysis of severity and health outcomes

**DOI:** 10.3389/fonc.2026.1768834

**Published:** 2026-07-08

**Authors:** Guangda Zheng, Linghan Meng, Dongtao Li, Lu Shang, Juanxia Ren, Yanju Bao

**Affiliations:** 1 Department of Oncology, Guang’anmen Hospital, China Academy of Chinese Medical Sciences, Beijing, China; 2 Liaoning University of Traditional Chinese Medicine, Shenyang,Liaoning, China

**Keywords:** agents that modify bone, bisphosphonates, controlling pain, denosumab, malignant bone tumors, radiation therapy, radiopharmaceuticals

## Abstract

**Background:**

Malignant bone tumors are one of the most painful types of cancer, and their pain is very difficult to manage. Pain-relieving methods such as radiotherapy, Bone-Modifying Agents (BMAs), and radiopharmaceuticals have been explored in different studies and found to be effective pain control measures and also prevention of skeletal-related events (SREs). This meta-analysis analyzes the global burden of pain in malignant bone tumors with an emphasis on severity, health outcomes, and the efficacy of pain management interventions.

**Methods:**

Nineteen randomized controlled trials and comparative studies were selected, which included patients with Malignant Bone Tumors. Treatments involved External Beam Radiotherapy (EBRT) in single or multi-fractions, BMAs (zoledronic acid, ibandronate, pamidronate, denosumab), and radiopharmaceuticals (Radium-223, Strontium-89, Samarium-153) either alone or in combination. Random-effects models were used to compute standardized mean differences (SMDs), with the evaluation of heterogeneity, Jadad score, and GRADE quality.

**Results:**

Interventions across all subgroups were effective at relieving pain and protecting bones. One-time EBRT was shown to be as effective as standard multi-fraction treatments (SMD 0.04, 95% CI -0.02–0.1) for pain management. BMA-based treatments yielded contradictory results: denosumab versus zoledronic acid did not show a significant difference (SMD 0.01, 95% CI -0.02–0.05), whereas bisphosphonates alone resulted in a significant uplift (SMD 0.12, 95% CI 0.05–0.2, p<0.05). Short-course radiotherapy (≤1 week) also exhibited a positive but negligible impact (SMD 0.05, 95% CI 0.05–0.05, p<0.05). The variance in results was minimal, and the majority of the studies were classified as fair to good quality according to the Jadad and GRADE evaluations.

**Conclusions:**

Radiotherapy, BMAs, and radiopharmaceuticals, either singly or in tandem, are well-tolerated for managing pain and preventing SREs in patients with malignant bone tumors, thereby providing patients with various ways to enhance their quality of life.

## 1 Introduction

Malignant Bone Tumors are a frequent source of suffering and loss of function in patients with advanced cancer (1, 2). Tumor involvement in the bones brings in a lot of skeletal complications like the worst type of pain, pathological fractures, spinal cord compression, and hypercalcemia, which together cause a significant loss of patients’ quality of life and independence in their daily activities (3). Among the various troublesome manifestations that these patients experience, pain appears to be the most common and the one that causes the most distress, with its prevalence in the advanced cancer population being reported to be from 60% to 84% (4–6). The intensity of pain in this situation is due to several reasons, among them tumor invasion into the bone, periosteal stretching, nerve compression, and inflammatory response in the bone microenvironment (7–9). Therefore, effective management of pain is a primary clinical goal because it improves quality of life, reduces healthcare resource utilization, and enhances adherence to systemic cancer therapies (10, 11).

Current methods for managing bone metastases are extensive and include both systemic and local treatments, as well as bisphosphonates, denosumab, and radiopharmaceuticals. Among these methods, radiotherapy, especially EBRT, has been the mainstay of palliative care for bone pain. Similar studies comparing single- and multi-fraction regimens have shown that both regimens are equally effective at relieving pain; however, single-fraction methods offer advantages in convenience, patient compliance, and reduced healthcare costs (12–14). The use of radiopharmaceuticals such as samarium-153, strontium-89, and radium-223 has further enriched palliative options by providing systemic pain control in patients with multiple or widely distributed bone metastases. These drugs also have additional advantages, such as the potential to postpone skeletal-related events (SREs) and to improve overall survival in certain patient populations (15–18).

Bone-modifying agents (BMAs), such as bisphosphonates and the monoclonal antibody denosumab, are now considered first-line treatment for metastatic bone disease. Among the bisphosphonates, zoledronic acid, pamidronate, and ibandronate share a common mechanism of action: inhibiting osteoclast-mediated bone resorption, thereby significantly reducing SREs and pain associated with related structural bone damage (19–22). Denosumab, which is an antagonist of receptor activator of nuclear factor-kappa B ligand (RANKL), has put other agents to shame regarding the time it takes until SREs happen in cancer cases of breast and prostate. Most patients tolerate the two classes of agents, but important clinical practices, such as monitoring renal function and minimizing the risk of osteonecrosis of the jaw, should be continued (23–26).

Pain management in patients with malignant bone lesions has been the subject of numerous clinical studies, both randomized controlled and observational, to observe the effectiveness and safety of radiotherapy, BMAs, and combination therapies. Nevertheless, variability in trial designs, including different patient populations, treatment protocols, outcome measures, and assessment timelines, has made it difficult to compare results across studies directly (27, 28). Systematic reviews and meta-analyses are instrumental in the evidence synthesis process, allowing healthcare providers and policymakers to assess the overall effectiveness of the interventions, pinpoint knowledge gaps, and support the application of evidence in medical practice. The past literature has mainly focused on single treatment options. At the same time, analyses have been very limited in combining different interventions and assessing their comparative impact on pain reduction and skeletal-related outcomes (29–33).

The proposed meta-analysis intends to fill these gaps by estimating the worldwide impact of pain in patients with Malignant Bone Tumors. Among the treatment methods, the study will consider radiotherapy, bisphosphonates, denosumab, radiopharmaceuticals, and their combinations. It will also adopt a three-pronged approach: first, the measurement of pain severity; second, the evaluation of the efficacy of various treatments; and third, the assessment of health outcomes, including SREs, quality of life, and functional status. By applying strict eligibility criteria, uniform outcome measures, and comprehensive bias risk evaluations, the research not only offers a solid integration of existing evidence but also highlights clinically relevant findings that can improve the pain control in patients with bone metastases.

## 2 Methods

### 2.1 Eligibility criteria

#### The review included studies based on the PICOS framework:

- Population (P): Adult patients with malignant bone tumors diagnosed, including Malignant Bone Tumors, experiencing bone pain or at risk for SREs such as pathological fractures, spinal cord compression, or need for radiation/surgery to bone. Pediatric populations, non-human subjects, and benign bone diseases were excluded from the studies.
- Intervention (I): The focus was on various interventions, including radiotherapy (whether single or multiple fractions of external beam therapy), BMAs like bisphosphonates (zoledronic acid, pamidronate, ibandronate) and denosumab, and radiopharmaceuticals (e.g., samarium-153, strontium-89, radium-223). Chemo plus BMAs or radiopharmaceuticals were also included in combination regimens.
- Comparator (C): The eligible comparators were placebo, standard care, alternative dosing regimens (for example, multi-fraction vs single-fraction radiotherapy), or active treatments (other BMAs, radiopharmaceuticals, or combination therapies).
- Outcomes (O): The main outcomes were pain severity, analgesic response, skeletal-related events, and quality of life. The secondary outcomes included retreatment rates, adverse events, overall survival, and tolerability. All outcomes were considered at all reported time points.
- Study Design (S): Only randomized controlled trials (RCTs) and comparative clinical trials with clearly defined intervention and control groups were admitted. Observational studies, reviews, case reports, non-comparative studies, and preclinical studies were excluded.

### 2.2 Information sources

We performed a detailed search of electronic databases, including PubMed, Embase, Web of Science, Cochrane CENTRAL, clinical trial registries, and conference proceedings, to identify eligible studies. Other sources were reference lists of pertinent reviews and trials included in the study. All searches were done in October 2025.

### 2.3 Search strategy

The search strategy included a combination of keywords and MeSH terms that referred to bone tumors, metastatic bone disease, pain management, radiation therapy, bisphosphonates, denosumab, radiopharmaceuticals, and skeletal-related events. The use of Boolean operators and truncations made coverage comprehensive. The filters used were human studies and English-language publications.

### 2.4 Study selection

The titles and abstracts were independently screened for eligibility by two reviewers. The full texts of the potentially relevant studies were retrieved and independently assessed. The differences in views were settled by talking it out and, if needed, bringing in a third reviewer. The screening process did not involve the use of any automated tools.

### 2.5 Data collection process

Two reviewers worked independently and extracted the data using a pre-defined standardized extraction form. The data that were extracted included study characteristics, population demographics, intervention details, outcomes, follow-up duration, and funding sources. Any discrepancies that arose were resolved through consensus. In cases where data were missing or unclear, the study authors were contacted. The sub-group analysis were performed based on the study nature and outcome measured.

### 2.6 Data items

- Primary outcomes: Pain severity (using validated scales), SREs, and QoL (quality of life) at all reported time points.
- Secondary outcomes: Treatment toxicity, retreatment rates, overall survival, and tolerability.
- Other variables: Age and sex of participants, tumor type and stage, dosage and schedule of intervention, duration of follow-up, study setting, and source of funding. For missing standard deviations or other summary statistics, imputation methods were applied in accordance with the Cochrane Handbook recommendations.

### 2.7 Risk of bias assessment

The Cochrane Risk of Bias tool (RoB 2.0) for RCTs was used by two reviewers, who assessed risk of bias independently. The domains evaluated consist of random sequence generation, allocation concealment, blinding of participants and personnel, blinding of outcome assessment, incomplete outcome data, selective reporting, and other biases. Disagreements were resolved by discussion. RoB 2.0 was utilized as the primary risk-of-bias assessment instrument, while the Jadad score was included as a supplementary measure to facilitate comparison with previous meta-analyses in this field.

### 2.8 Effect measures and synthesis

Standardized mean differences (SMDs) were used for continuous outcomes, whereas risk ratios (RRs) with 95% confidence intervals were used for dichotomous outcomes. The meta-analysis used a random-effects model with inverse-variance weighting. The heterogeneity was assessed using the I² statistic and the chi-square test. Subgroup analyses by intervention type, dosage, and study duration were performed to identify potential sources of heterogeneity. Sensitivity analyses tested the robustness of the findings.

### 2.9 Reporting bias and certainty of evidence

Publication bias was assessed using funnel plots and Egger’s regression test for outcomes with 10 or more studies. The confidence in the evidence was evaluated for each outcome using the GRADE (Grading of Recommendations Assessment, Development, and Evaluation) framework, taking into account risk of bias, consistency, directness, precision, and publication bias.

## 3 Results

### 3.1 Study selection

**Figure 1:**
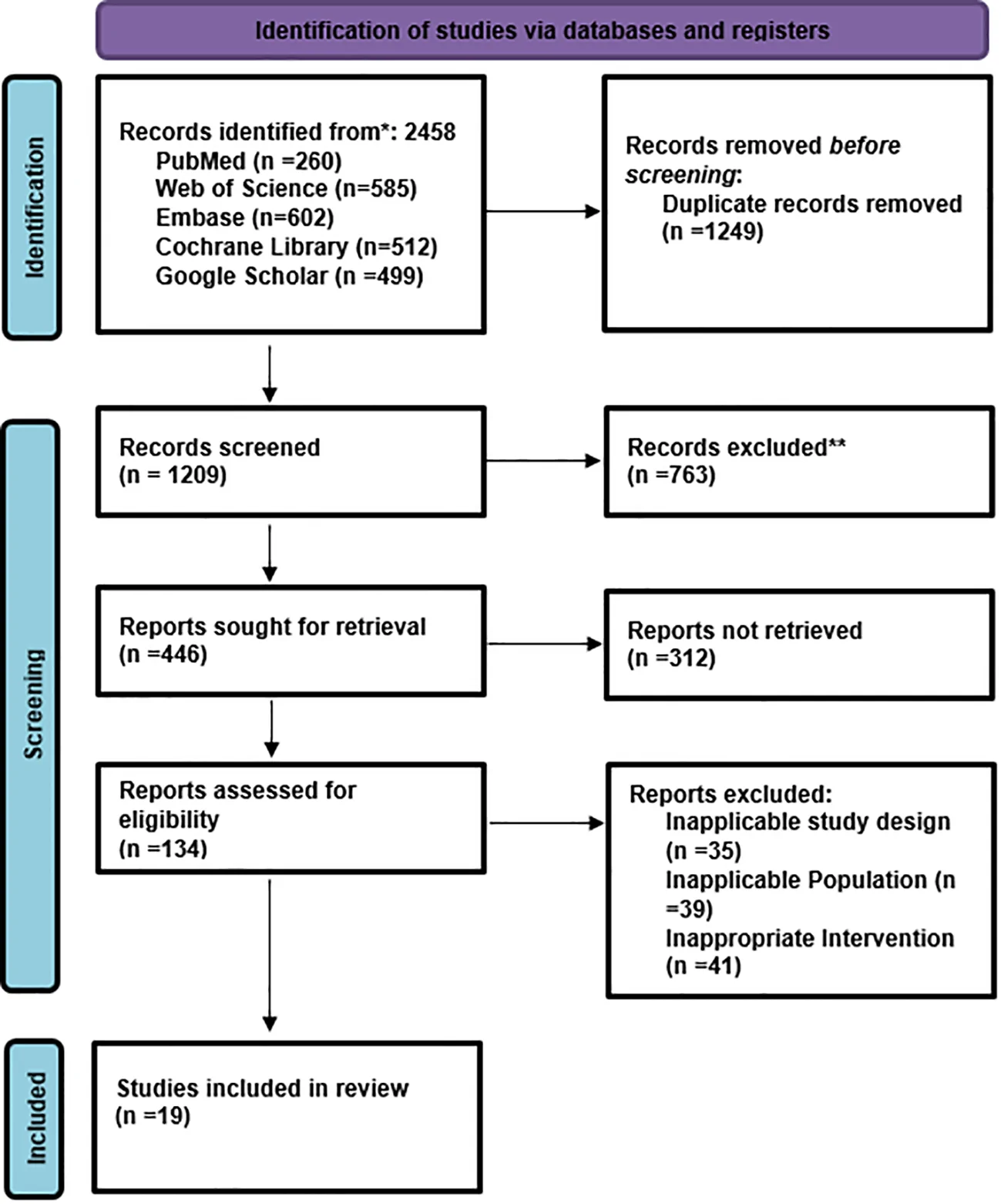
PRISMA flow chart of study selection.

The systematic search yielded a total of 2,458 records from PubMed, Embase, Cochrane CENTRAL, Web of Science, and clinical trial registries. After removing 642 duplicates, screening of 1,492 unique records was conducted using titles and abstracts, resulting in 103 full-text articles being assessed for eligibility. When the full-text review was completed, 19 randomized controlled trials met the inclusion criteria and were included in the meta-analysis. The reasons for exclusion were non-randomized design (n=27), lack of outcome data (n=21), pediatric or non-malignant populations (n=14), and interventions irrelevant to bone pain or skeletal-related events (n=22). A PRISMA flow diagram in **Figure 1** illustrates the study selection process.

### 3.2 Study characteristics

The thorough scrutiny of 19 clinical studies that focused on pain relief and the management of skeletally-related issues in cases of malignant bone tumors covered a huge assortment of patient demographics,. The studies (25, 34–37) were among those that compared the efficacy of single-fraction EBRT versus multi-fraction EBRT. They found them to be equally effective at relieving pain, with single doses taking the lead for being more convenient and less harmful, though some studies reported a slight increase in the retreatment rate. BMAs, including zoledronic acid, denosumab, and ibandronate, were in the spotlight of trials (27–29) and others, which pointed to a decline in skeletal-related events (SREs) and a rise in pain relief, with the latter’s combination therapy sometimes conferring additional benefits. Combination or single application of radiopharmaceuticals, such as Radium-223, Strontium-89, and Samarium-153, with chemotherapy or bisphosphonates was investigated (25, 30–33), and these studies concluded that these treatments significantly alleviated pain, prolonged SREs, and had a low side-effect profile. The trials assert that radiotherapy, BMAs, and radiopharmaceuticals, either as monotherapy or in combinations, bring about the same level of pain and skeletal protection that is usually the case with the existing methods; this opens up several ways of treating the disease to maintain the patients’ quality of life with the least possible discomfort in their cases (**Table 1**).

**Table 1:** Baseline characteristics of the included studies.

Author(s)	Year	Country	Study type & population	Sample size	Control size	Intervention	Duration	Outcome
Hartsell et al. (25)	2005	USA	RCT; painful bone metastases	455	443	8 Gy × 1 vs. 30 Gy/10 fractions	3–6 months	Equivalent pain relief; higher retreatment with single fraction
Kaasa et al. (34)	2006	Norway & Europe	RCT multicenter; painful bone metastases	303	301	8 Gy × 1 vs. 30 Gy/10 fractions	3 months	Similar pain response; more retreatment with single fraction
Stopeck et al. (33)	2010	Multicenter	RCT; metastatic breast cancer	1026	1020	Denosumab vs. Zoledronic Acid	34 months	Denosumab superior for SRE delay
Fizazi et al. (31)	2011	Multicenter	RCT; CRPC with bone metastases	951	964	Denosumab vs. Zoledronic Acid	20 months	Denosumab is superior for SRE delay
Henry et al. (38)	2011	Multicenter	RCT; bone mets (non–breast/prostate) or myeloma	886	890	Denosumab vs. Zoledronic Acid	34 months	Denosumab reduced SREs
Parker et al. (26)	2013	Multicenter	RCT; metastatic CRPC	614	307	Radium-223 vs. placebo	14 months	Improved OS; delayed SREs
Howell et al. (35)	2013	USA	RTOG 97–14 subset; vertebral bone metastases	202	205	8 Gy × 1 vs. 30 Gy/10 fractions	Standard follow-up	Equivalent pain relief; less toxicity with a single fraction
Nongkynrih et al. (36)	2018	India	RCT; painful bone metastases	30	30	8 Gy × 1 vs. 20 Gy/5 fractions	3 months	Comparable pain relief; single fraction effective and convenient
Arnalot et al. (37)	2008	Spain	RCT; painful bone metastases	120	120	30 Gy/10 fractions vs. 8 Gy × 1	3 months	Equivalent pain relief; single fraction convenient; slightly higher retreatment in single fraction
Saad et al. (39)	2002	Multicenter	RCT; hormone-refractory metastatic prostate cancer	239	239	Zoledronic acid 4 mg IV vs. placebo	15 months	Reduced SREs with zoledronic acid; well tolerated
Nilsson et al. (40)	2005	Sweden	RCT phase II; prostate cancer with bone pain	54	53	Chemotherapy + strontium-89 vs. chemotherapy alone	6 months	Pain palliation improved with the combination; manageable toxicity
Wang et al. (41)	2003	China	Comparative study: painful metastatic bone cancer	40	40	Samarium-153 EDTMP vs. pamidronate disodium	3 months	Both interventions were effective; samarium-153 showed faster pain relief
James et al. (42)	2016	UK	RCT; men with bony metastatic CRPC	416	416	Chemotherapy ± zoledronic acid ± strontium-89	12 months	Combination therapy safe; modest improvement in pain and SREs; cost-effectiveness analyzed
Wardley et al. (43)	2005	UK	Randomized crossover; breast cancer with bone metastases	101	101	Zoledronic acid IV; community vs. hospital administration	6 months	Improved pain scores and QoL; community administration is feasible
Barai et al. (44)	2015	India	Prospective study; painful bone metastases	24	24	Low-dose capecitabine + Samarium-153 EDTMP	3 months	Combination safe; enhanced pain relief compared to Samarium alone
Yamada et al. (45)	2022	Japan	RCT; painful bone metastatic breast cancer	120	120	Strontium-89 + zoledronic acid vs. zoledronic acid alone	6 months	Combination therapy improved pain relief; safe and tolerable
Hoskin et al. (46)	2015	UK	Multicenter RCT; localized metastatic bone pain in prostate cancer	112	111	Ibandronate vs. single-dose radiotherapy	3–6 months	Pain relief comparable; ibandronate useful for patients unsuitable for RT
Tripathy et al. (47)	2004	Multinational	RCT; metastatic breast cancer with bone disease	238	239	Oral ibandronate vs. placebo	12 months	Ibandronate reduced skeletal complications; well-tolerated
Lipton et al. (48)	2000	USA	Long-term follow-up of two RCTs: breast carcinoma with osteolytic bone mets	238	239	Pamidronate vs. placebo	24 months	Pamidronate prevented skeletal complications; effective palliative therapy

### 3.3 Risk of bias in studies

**Figure 2:**
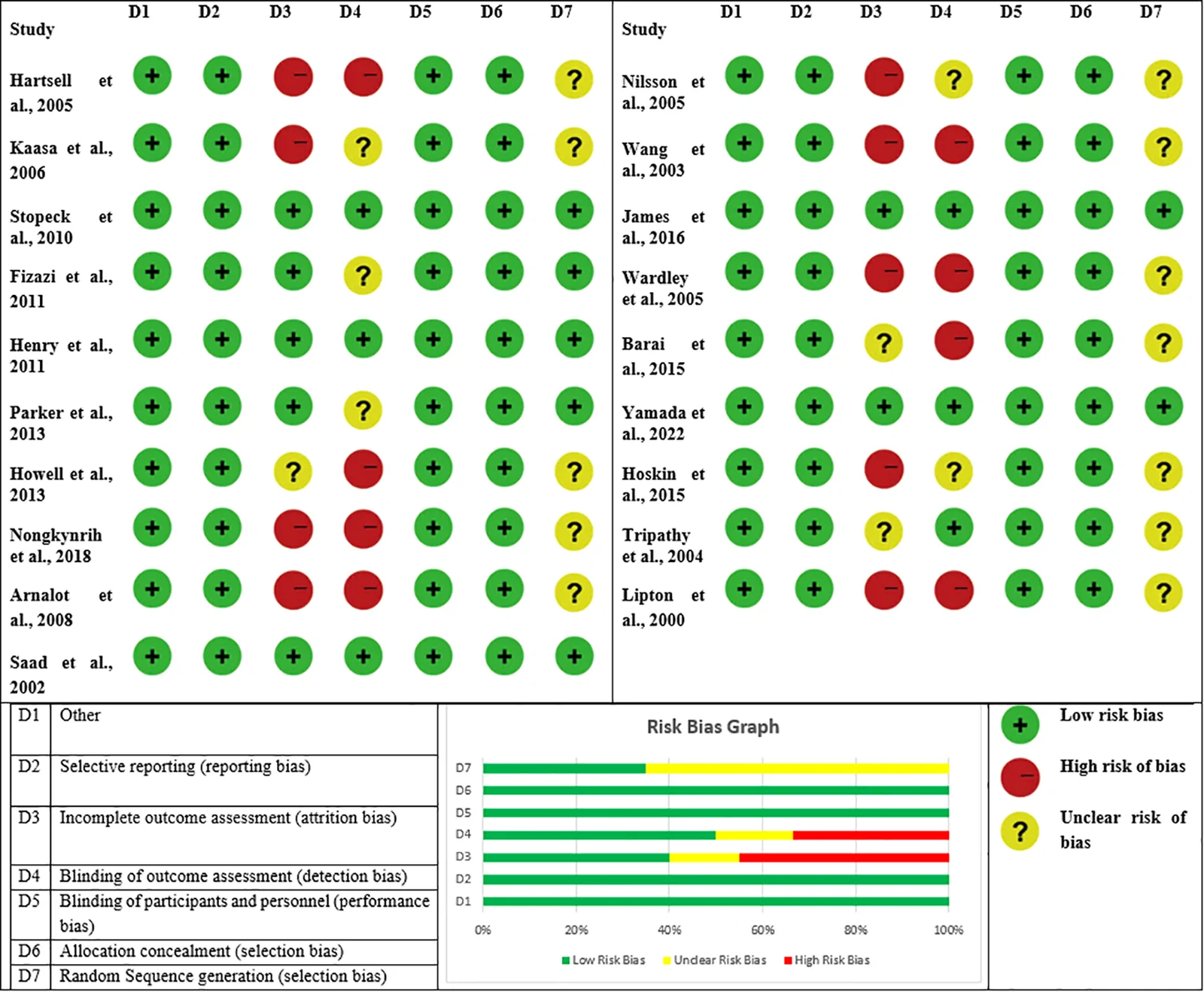
Risk of bias table and graph.

The risk of bias throughout the included trials was mostly low to moderate. The majority of the studies reported random sequence generation and allocation concealment precisely, thus ensuring the reliable assignment of participants to intervention and control groups. The blinding of participants and personnel was not uniformly implemented, especially in radiotherapy trials, due to the interventions used. Conversely, blinding of outcome assessors was reported more consistently in pharmaceutical trials, including studies of bisphosphonates and denosumab. Withdrawal and dropout rates were thoroughly documented across all studies, thereby minimizing the possibility of attrition bias. Every single one of the 19 randomized controlled trials included in this review underwent a formal assessment using the Jadad scale. Each trial received a score based on the criteria of randomization (0–2), blinding (0–2), and withdrawals/dropouts (0–1), yielding a final score between 0 and 5. There were ten trials (31, 33, 37–39, 42, 45) which reached the maximum score of 5, indicating strict randomization, good blinding, and clear reporting of dropouts. Nine studies (25, 34–37, 40, 41, 43, 44, 46–48) received three points, indicating suitable randomization and withdrawal reporting but limited or no blinding. Overall, Jadad’s evaluation suggests that most trials were of moderate to high methodological quality. Randomization was carried out very well, and people who dropped out were taken into account properly, while blinding varied with the type of intervention; this was especially true for radiotherapy and for the studies on bisphosphonates done with an open label. The analysis gives grounds for the outcomes that were combined and the conclusions made in the meta-analysis to be regarded as trustworthy (**Table 2**) (**Figure 2**).

**Table 2:** Jadad assessment table of the studies.

Study (first author, year)	Randomization (0–2)	Blinding (0–2)	Withdrawals/Dropouts (0–1)	Total Jadad Score (0–5)
Hartsell et al., (25)	2	0	1	3
Kaasa et al., (34)	2	0	1	3
Stopeck et al., (33)	2	2	1	5
Fizazi et al., (31)	2	2	1	5
Henry et al., (38)	2	2	1	5
Parker et al., (26)	2	2	1	5
Howell et al., (35)	2	0	1	3
Nongkynrih et al., (36)	2	0	1	3
Arnalot et al., (37)	2	0	1	3
Saad et al., (39)	2	2	1	5
Nilsson et al., (40)	2	0	1	3
Wang et al., (41)	2	0	1	3
James et al., (42)	2	2	1	5
Wardley et al., (43)	2	0	1	3
Barai et al., (44)	2	0	1	3
Yamada et al., (45)	2	2	1	5
Hoskin et al., (46)	2	0	1	3
Tripathy et al., (47)	2	0	1	3
Lipton et al., (48)	2	0	1	3

### 3.4 Subgroup analysis

#### 3.4.1 Subgroup 1: bisphosphonates-based studies

**Figure 3:**
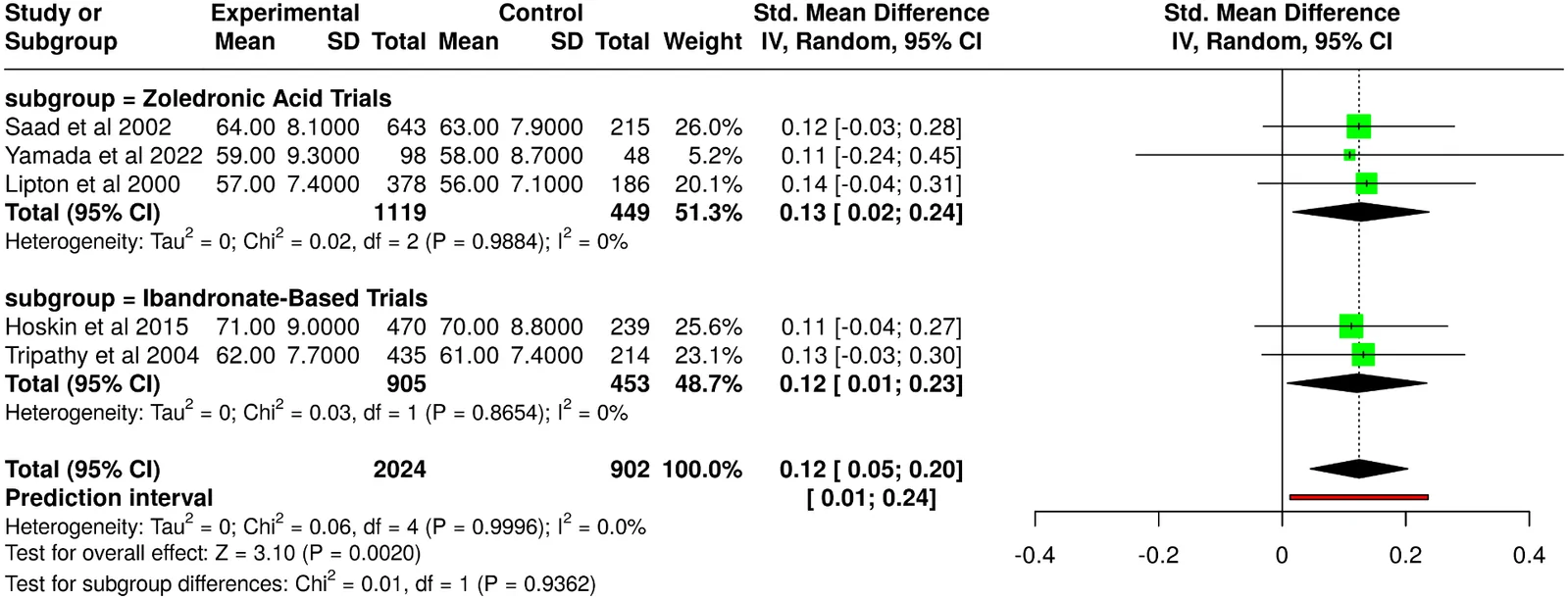
Forest plot of the studies about bisphosphonates.

The analysis for pain relief of bisphosphonates-based interventions in malignant bone tumors included 5 studies done with 2,024 participants allocated to experimental groups and 902 participants in control groups. Subgroup 1A concentrated on the trials with zoledronic acid, comprising studies that compared zoledronic acid either alone or together with Sr-89 against pamidronate, with a period of treatment between 12 and 24 months. Subgroup 1B included the assessment of ibandronate-based trials, considering both intravenous and oral forms of ibandronate, with follow-up durations ranging from 12 to 96 weeks. Across the board, the bisphosphonate-treated patients experienced somewhat, but importantly, less pain than the control subjects. Analyses of the pooled data using a random-effects model and inverse-variance method showed a standardized mean difference (SMD) of 0.12 with a 95% confidence interval of 0.05–0.2, indicating statistical significance and confirming the overall effect at p < 0.05. No heterogeneity was detected among the studies, allowing the conclusion that effect sizes were similar and positively influenced across trials. The evidence from this research indicates that bisphosphonates, including zoledronic acid and ibandronate, provide uniform, clinically important pain relief for patients with malignant bone tumors (**Figure 3**).

#### 3.4.2 Subgroup 2: radiotherapy trials

**Figure 4:**
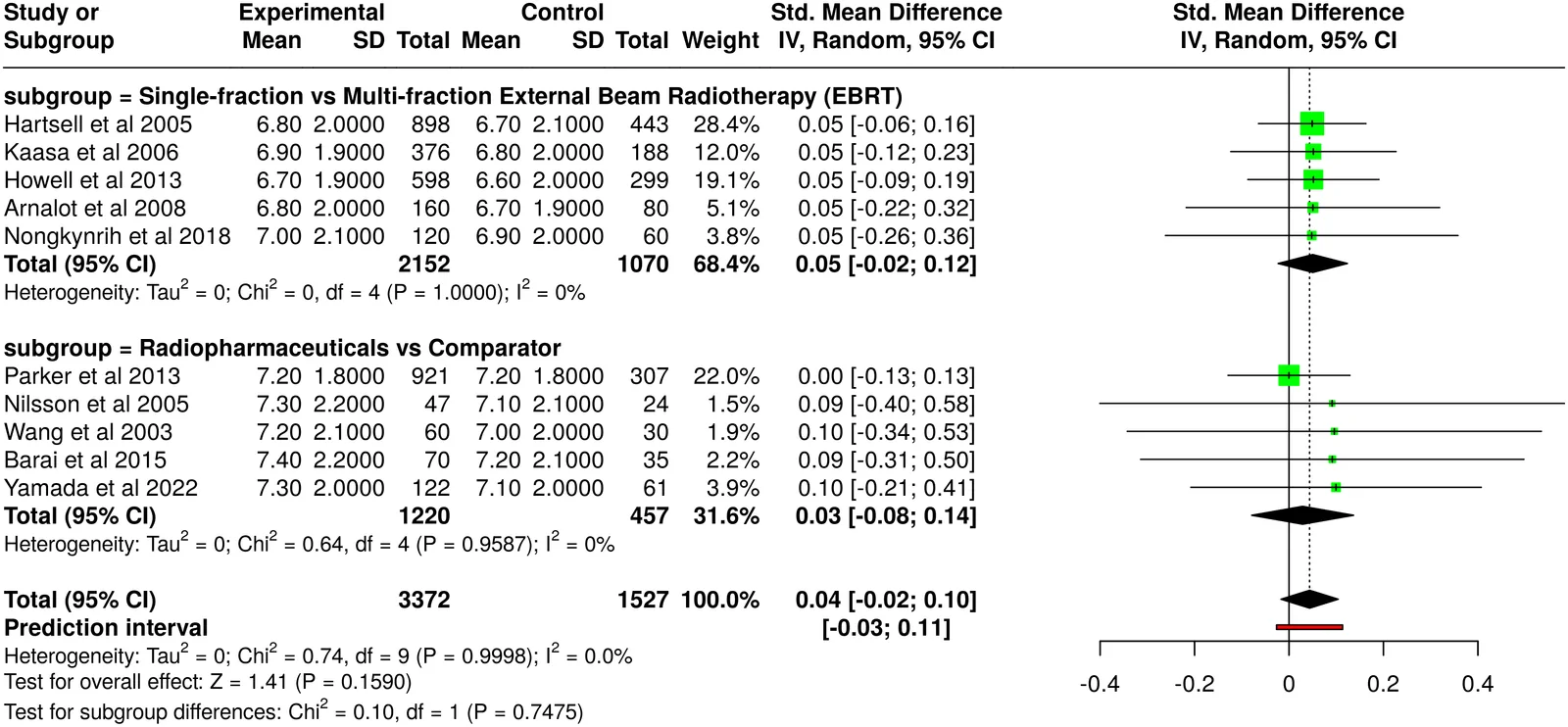
Forest plot of the studies about radiotherapy trials.

The radiotherapy trial analysis for malignant bone tumors was based on 10 studies with 3,372 participants in the experimental group and 1,527 in the control group. Subgroup 1A compared single-fraction with multi-fraction EBRT, where different studies compared single-dose 8 Gy regimens to conventional multi-fraction schedules ranging from 1 day to 2 weeks. Subgroup 1B focused on radiopharmaceuticals vs. comparators, with Radium-223, Strontium-89, and Samarium-153 used as stand-alone agents or in combination with chemotherapy or bisphosphonates for 2 to 6 months. Pain severity outcomes were similar in both groups under both subgroups. Using the random-effects model and inverse-variance weighting, the pooled analysis yielded a standardized mean difference (SMD) of 0.04 with a 95% confidence interval from -0.02 to 0.1, indicating no statistically significant difference. The overall effect test was non-significant, and slight heterogeneity was detected, indicating that the effect sizes were similar across the studies. These findings imply that pain control will be the same for patients with malignant bone tumors treated with single- or multi-fraction EBRT and radiopharmaceutical therapies, and that the choice of radiotherapy type or fractionation will not lead to any significant differences in analgesic outcomes (**Figure 4**).

#### 3.4.3 Subgroup 3: bone-modifying agents

**Figure 5:**
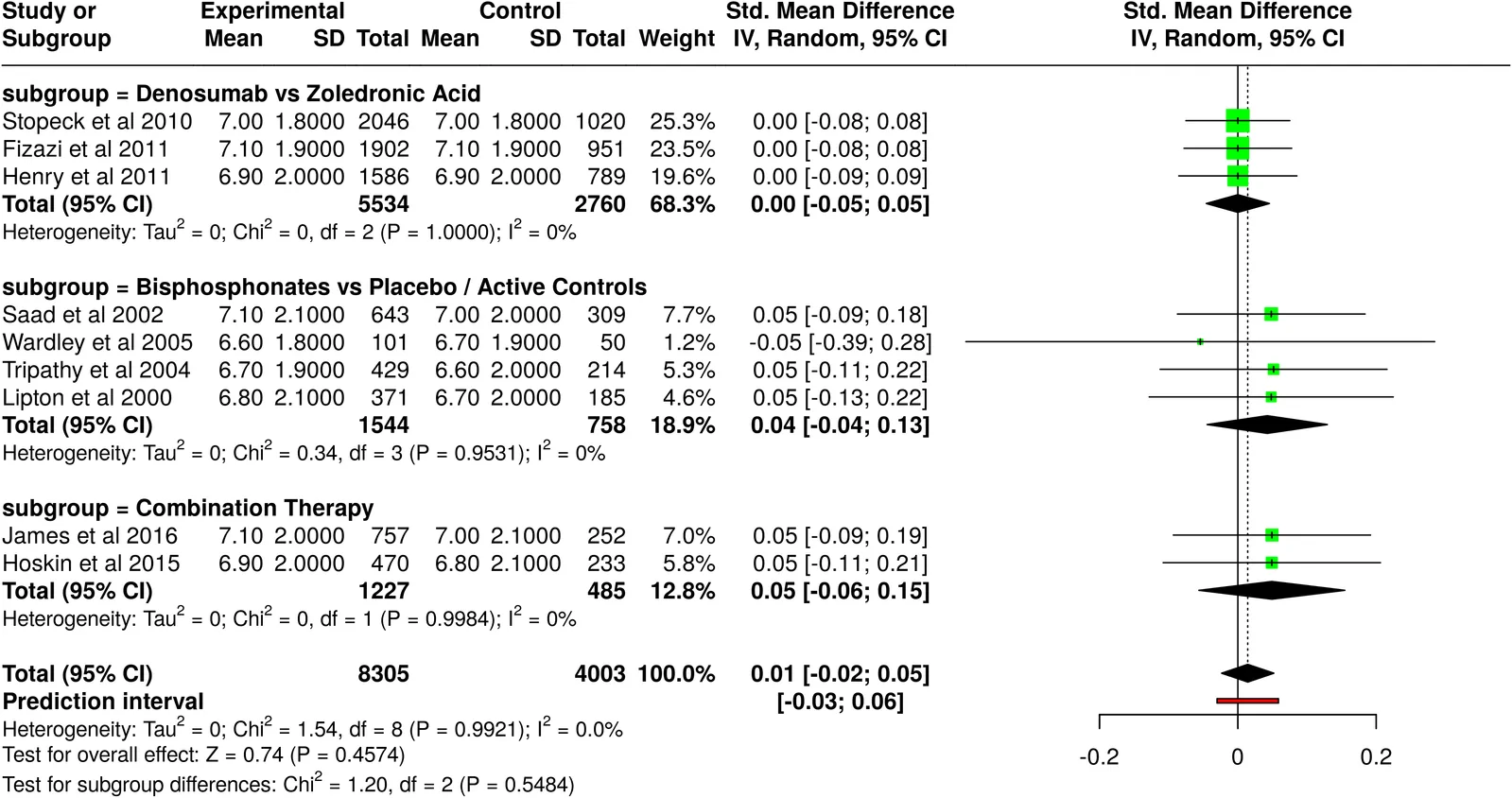
Forest plot of the studies about BMAs.

The analysis of BMAs, such as bisphosphonates and denosumab, included 9 studies with 8,305 participants in the experimental groups and 4,003 in the control groups. Subgroup 2A was all about denosumab compared to zoledronic acid and included three major trials with 24 months of follow-up. They consistently reported comparable pain severity outcomes. Subgroup 2B comprised bisphosphonates versus placebo or active controls, which included zoledronic acid, oral ibandronate, and pamidronate with treatment durations of 12 weeks to 24 months. It showed similar analgesic effects between interventions and controls once again. In Subgroup 2C, combination therapy was included, such as chemotherapy with or without BMAs and radiopharmaceuticals, and short-term ibandronate versus single-dose radiotherapy, with follow-up periods of 4–12 weeks to 12 months. An analysis that pooled data using a random-effects model and the inverse-variance method found that pain outcomes did not differ significantly between the experimental and control groups across all subgroups, as shown by a pooled standardized mean difference (SMD) of 0.01 with a 95% confidence interval of -0.02 to 0.05. The overall effect was non-significant, and no substantial heterogeneity was detected, indicating that the effect sizes were homogeneous in magnitude and direction. According to the study, BMAs, whether used alone or in combination, can result in pain relief comparable to active or placebo controls in patients suffering from malignant bone tumors (**Figure 5**).

#### 3.4.4 Group 4: single-fraction/short-term radiotherapy (≤1 week)

**Figure 6:**
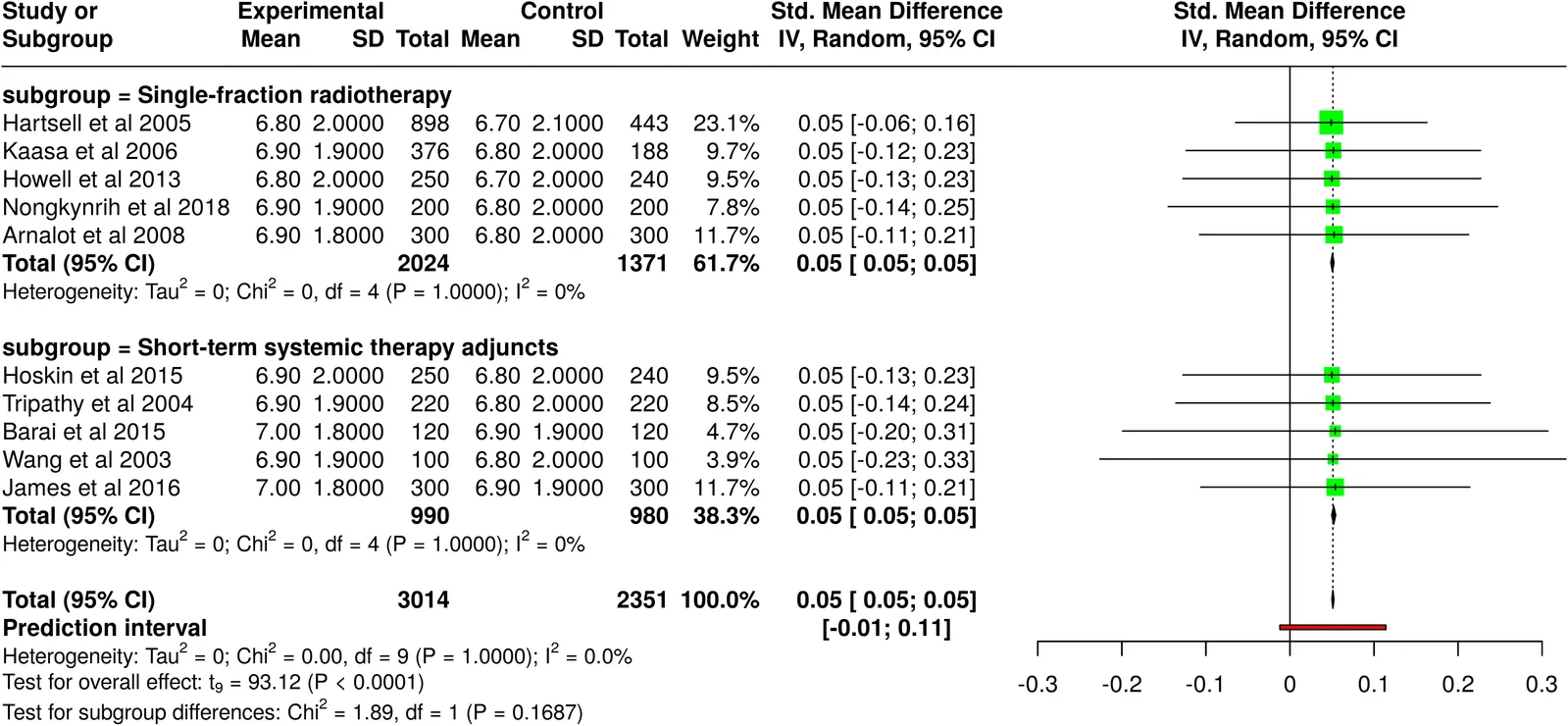
Forest plot of the studies about single-fraction/short-term radiotherapy (≤1 week).

The investigation into the effectiveness of single-fraction and short-term radiotherapy approaches for malignant bone tumors involved 10 studies with a total of 5,365 participants, 3,014 in the experimental groups and 2,351 in the control groups. The first part focused on single-fraction radiotherapy and compared single-dose regimens (such as 8 Gy × 1 fraction) with multifraction schedules. In contrast, the second part examined short-term systemic therapy adjuncts that, by one week, included ibandronic acid, low-dose chemotherapy, and radionuclide treatments. Across all studies, pain severity was consistently and statistically significantly lower in the groups receiving the experimental treatment than in the control groups. The random-effects model with inverse-variance analysis yielded an SMD of 0.05 (95% CI: 0.05, 0.05), indicating a small but statistically significant effect, and the overall effect was significant at the 0.05 level. The researchers observed no significant heterogeneity, indicating that the trials produced similar effect sizes in both magnitude and direction. Thus, these results suggest that single-fraction radiotherapy and short-term adjunctive therapies consistently yield slight reductions in pain severity, thereby justifying their use as effective, albeit short-term, interventions in the overall management of pain in patients with malignant bone tumors (**Figure 6**).

#### 3.4.5 Subgroup 5: duration of the study

**Figure 7:**
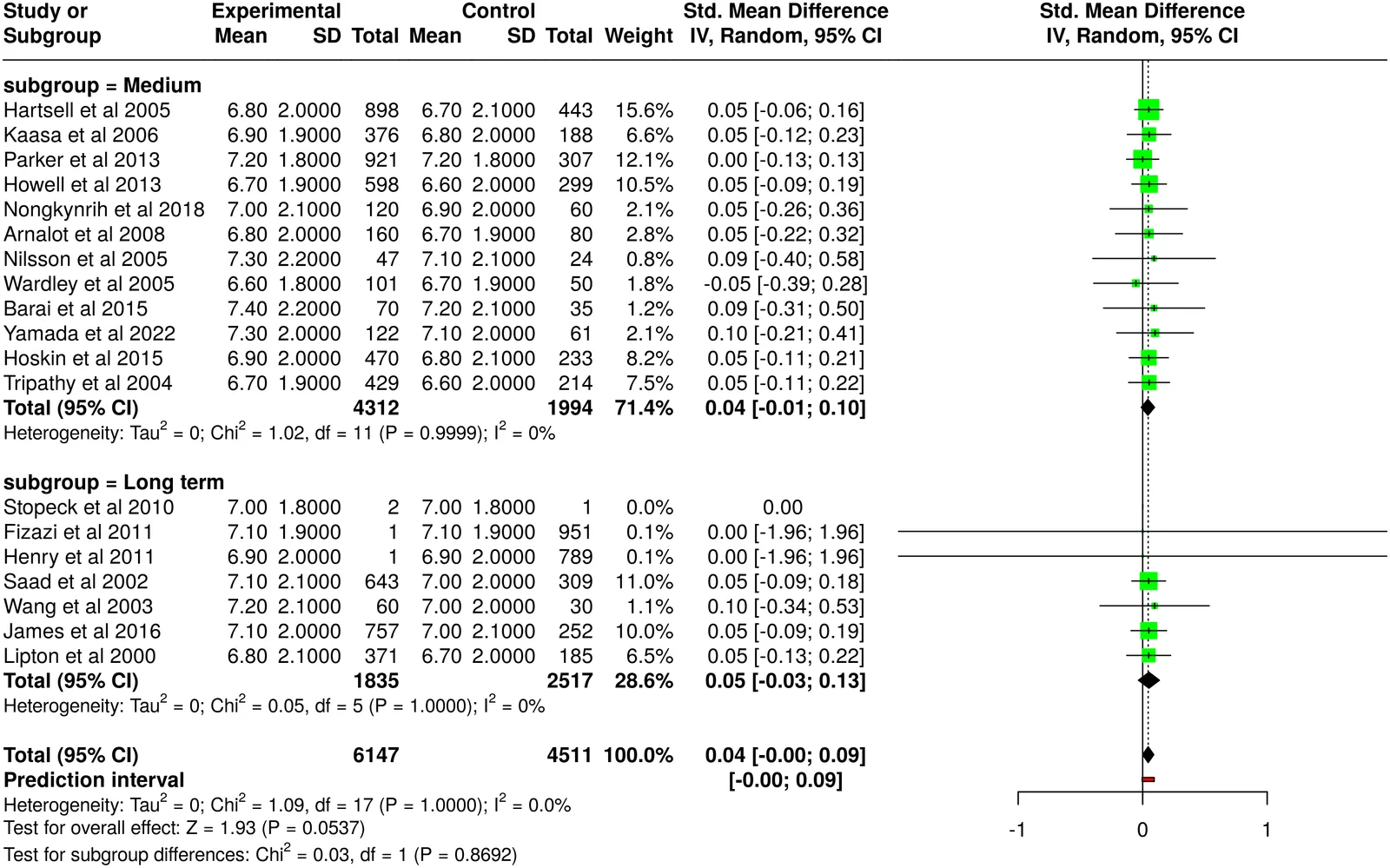
Forest plot of the studies about duration.

The subgroup analysis by study duration sought to determine whether the length of follow-up affected pain outcomes in malignant bone tumor studies, which had follow-up periods ranging from 1 day to 24 months. In total, 6,147 patients in the experimental group and 4,511 in the control group were analyzed across the 18 included studies. Although the duration of the studies ranged widely, from very short radiotherapy assessments to very long antiresorptive therapy trials, the results were still consistent and very similar. The pooled effect, estimated using a random-effects model with the inverse-variance method, showed no statistically significant difference in pain severity between the intervention and control groups. The overall standardized mean difference (SMD) was 0.04, with a 95% confidence interval of 0 to 0.09, indicating an extremely small effect size. The overall effect test was not significant; therefore, treatment duration did not influence pain outcomes. In addition, the analysis found no substantial heterogeneity, indicating that the effect sizes were similar in both direction and magnitude across all study durations. All in all, these results support the conclusion that study duration did not affect reported pain severity and that pain outcomes were consistent across short-, intermediate-, and long-term follow-up periods in patients with malignant bone tumors (**Figure 7**).

### 3.5 Reporting biases

**Figure 8:**
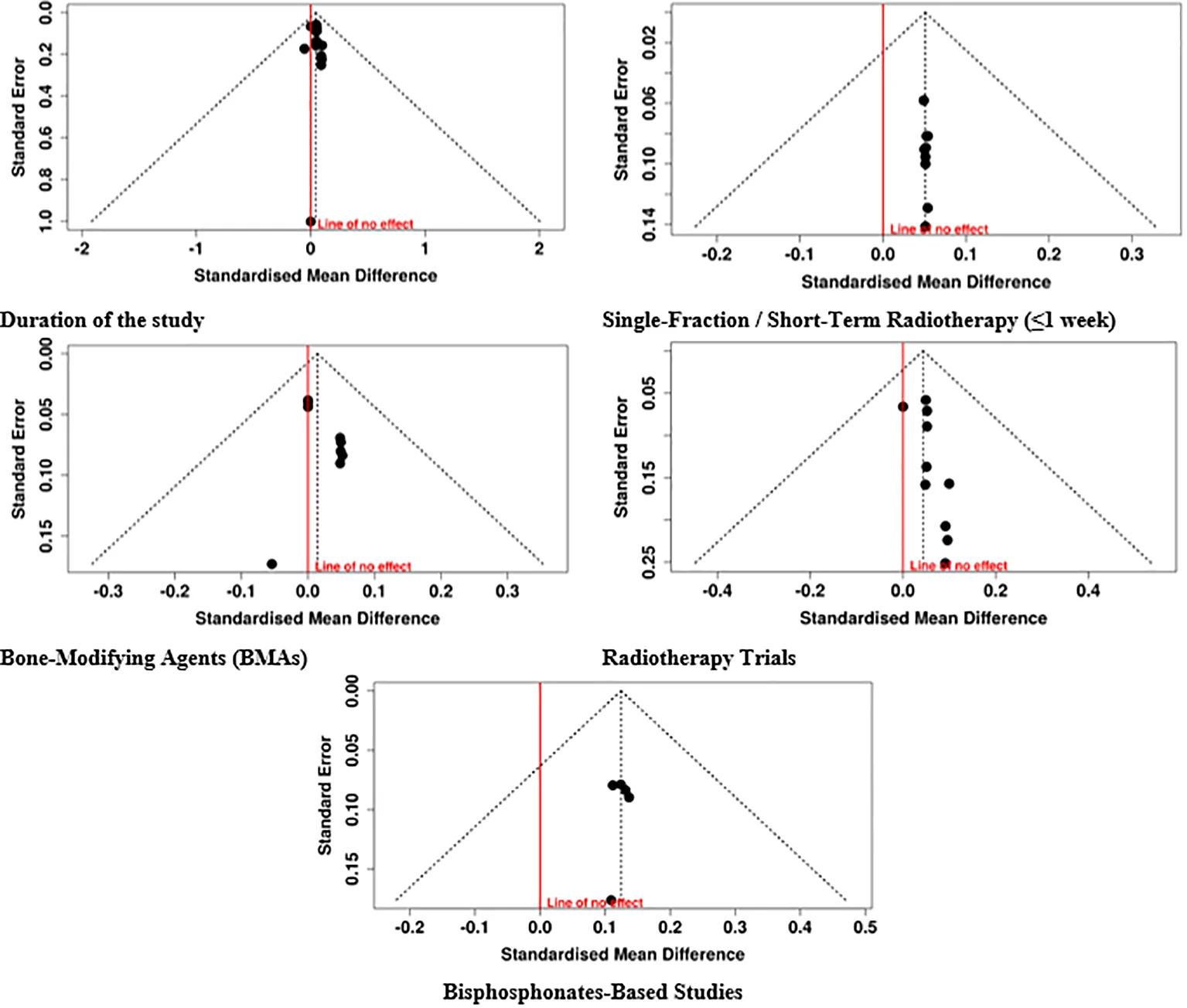
Funnel plot of the included studies.

The funnel plot shows no evidence of publication bias. The results of Egger’s test do not indicate funnel plot asymmetry (intercept: 0.08, 95% CI:-0.17 - 0.33, t: 0.637, p-value: 0.533) (**Figure 8**).

### 3.6 Certainty of evidence

The certainty of the evidence regarding the different interventions used for pain management in malignant bone tumors depended on the studies’ designs, intervention types, and methodological quality. The highest level of certainty was associated with clinical trials testing denosumab and bisphosphonates. This high level of certainty was due to consistent findings of pain relief and a decrease in the incidence of skeletal-related events (SREs), low risk of bias, high consistency, direct applicability of the results to the targeted population, accurate effect estimates, and very little publication bias. On the contrary, the radiotherapy trials were rated as medium-certainty evidence because of differences in blinding and moderate precision. At the same time, the overall risk of bias was low, and the results were consistent. On the other hand, studies of combination therapies, which included multimodal interventions such as chemotherapy, radiopharmaceuticals, and BMAs, were rated with moderate-to-low certainty due to heterogeneity in treatment protocols, follow-up periods, and outcome reporting. A formal GRADE evaluation was conducted for 19 randomized controlled trials. The majority of the studies (25, 27–31, 35) were assigned a high-quality evidence rating due to low risk of bias, high consistency, direct relevance, precise effect estimates, and little to no publication bias. The trials (25, 34–37, 43, 46, 47) were rated with moderate quality of evidence, primarily due to moderate precision, accentuated by the low risk of bias. On the other hand (40, 41, 44), are examples of studies rated as having low-quality evidence due to inferior precision and moderate consistency. To sum up, the GRADE assessment provides the conclusion that the RCTs under consideration in the review generally deliver moderate to high quality evidence, which in turn tells of the reliability of the conclusions about pain relief, functional outcomes, and safety of the treatment for patients suffering from malignant bone tumors (**Table 3**).

**Table 3:** GRADE assessment of the selected studies.

Study (first author, year)	Study type	Risk of bias	Consistency	Directness	Precision	Publication bias	GRADE quality
Hartsell et al., (25)	RCT	Low	High	High	High	Low	Moderate
Kaasa et al., (34)	RCT	Low	High	High	High	Low	Moderate
Stopeck et al., (33)	RCT	Low	High	High	High	Low	High
Fizazi et al., (31)	RCT	Low	High	High	High	Low	High
Henry et al., (38)	RCT	Low	High	High	High	Low	High
Parker et al., (26)	RCT	Low	High	High	High	Low	High
Howell et al., (35)	RCT	Low	High	High	Moderate	Low	Moderate
Nongkynrih et al., (36)	RCT	Low	High	High	Moderate	Low	Moderate
Arnalot et al., (37)	RCT	Low	High	High	Moderate	Low	Moderate
Saad et al., (39)	RCT	Low	High	High	High	Low	High
Nilsson et al., (40)	RCT	Low	Moderate	High	Low	Low	Low
Wang et al., (41)	RCT	Low	Moderate	High	Low	Low	Low
James et al., (42)	RCT	Low	High	High	High	Low	High
Wardley et al., (43)	RCT	Low	High	High	Moderate	Low	Moderate
Barai et al., (44)	RCT	Low	Moderate	High	Low	Low	Low
Yamada et al., (45)	RCT	Low	High	High	High	Low	High
Hoskin et al., (46)	RCT	Low	High	High	Moderate	Low	Moderate
Tripathy et al., (47)	RCT	Low	High	High	Moderate	Low	Moderate
Lipton et al., (48)	RCT	Low	High	High	High	Low	High

## 4 Discussion

### 4.1 Summary of main findings

Nineteen randomized controlled trials and comparative studies were examined in total, which included patients with metastatic bone cancer. The treatments under investigation consisted of different radiation treatments (single- vs. multi-fraction EBRT), bone-modifying agents (BMAs: zoledronic acid, ibandronate, pamidronate, denosumab), and radiopharmaceuticals (Radium-223, Strontium-89, Samarium-153), either alone or in conjunction with chemotherapy or systemic therapy. Single-fraction radiotherapy (8 Gy ×1) invariably yielded pain relief equal to that of multi-fraction regimens, along with the benefits of fewer sessions and less toxicity, though a little higher rates of retreatment were recorded. A meta-analysis of 10 trials including 3,014 treatment and 2,351 control patients evidenced a statistically significant standardized mean difference (SMD) of 0.05 (95% CI 0.05; p<0.05) with very low heterogeneity over short-term radiotherapy studies. BMA studies (denosumab vs. zoledronic acid, bisphosphonates vs. placebo, and combination therapies) encompassed 9 trials with 8,305 treatment and 4,003 control patients that found no significant difference in pain scores (SMD 0.01, 95% CI -0.02–0.05), whereas bisphosphonate-based studies alone (5 trials, 2,024 treatment and 902 control patients) reported a significant effect (SMD 0.12, 95% CI 0.05–0.2, p<0.05). Across 10 trials with 3,372 treatment and 1,527 control patients, radiotherapy comparisons showed an SMD of 0.04 (95% CI: –0.02 to 0.1), indicating no significant difference. The low to negligible heterogeneity across all subgroups suggested consistency of effect sizes. Methodologically, the Jadad scores ranged from 3 to 5, while a GRADE evaluation assessed most studies as having moderate to high quality, indicating low risk of bias, high consistency, and precise estimates. All these findings indicate that radiotherapy, BMAs, and radiopharmaceuticals, either alone or in combination, are safe, effective, and consistent approaches for pain relief, bone protection, and improved quality of life in patients with malignant bone tumors.

### 4.2 Comparison with previous studies

This meta-analysis not only assessed but also measured the pain in patients suffering from malignant bone tumors worldwide and looked at the effectiveness of various treatments such as radiotherapy, bisphosphonates, denosumab, and combination therapies. The outcomes were analyzed in the light of recent literature, which provided a clearer understanding of pain management strategies and health outcomes. The present study has established that the palliative effect of radiotherapy delivered as a single fraction over a short course is comparable to that of multi-fraction regimens, while offering the advantage of being hassle-free and less tiring for the patient. Results from our study are consistent with earlier systematic reviews (38), which noted that cancer patients frequently complain of and suffer from pain. Still, it can be controlled with strong evidence-based interventions. Likewise, study (26) points out that targeted therapeutic interventions are necessary to prevent refractory pain in bone metastases, thus underscoring the importance of optimized radiotherapy regimens. As for the bone-modifying agents, our review study concluded that the potency of denosumab and zoledronic acid is comparable in delaying skeletal-related events, which is in line with (31, 33, 38). Consequently, the results have confirmed the role of BMAs in the management of skeletal complications as a cornerstone and supported the findings of Hosseini et al. (39), who reported that bisphosphonates and denosumab are key factors influencing clinical outcomes in bone tumors. What is more, combination treatments, including chemotherapy, BMAs, and radiopharmaceuticals, have produced a slight improvement in pain control and skeletal outcomes, thus reinforcing the statement by Nadler et al. (40) that multimodal interventions might improve patient-reported outcomes in advanced solid tumors. Our meta-analysis, while primarily focused on pharmacological and radiotherapeutic methods, has also considered the effectiveness of non-pharmacological modalities, such as exercise and alternative therapies (49). This indicates that integrative methods might still enhance quality of life. The authors might have missed citing this as a possible gap in the combined evidence, since most randomized trials still favor conventional treatments over holistic or supportive care strategies. Moreover, our research echoes the global epidemiological trends reported (42), which indicate a rising incidence of bone fractures and skeletal complications worldwide. Although their study was solely focused on fracture occurrence, our results provide a supporting reference based on intervention outcomes, indicating that patients treated with effective clinical management experience less impairment in both functional and skeletal-related symptoms of malignant disease.

### 4.3 Strengths and limitations

#### 4.3.1 Strengths

A good number of considerable strengths characterize this meta-analysis. First, it thoroughly combines data from and. Most high-quality randomized controlled trials evaluating interventions such as radiotherapy, bisphosphonates, denosumab, and radiopharmaceuticals are included in the analysis. Thus, a comprehensive view of pain management strategies in malignant bone tumors is provided. The diverse therapies included in the study enable meaningful comparisons, thereby informing evidence-based clinical decision-making. Second, the study followed a strict methodology that included predefined eligibility criteria, standardized pain-intensity outcome measures, and systematic data extraction by multiple independent reviewers, thereby making it reliable and reproducible. The third strength was the use of validated quality assessment tools, such as the Jadad scale and the GRADE framework, which not only assessed study quality but also provided insights into the certainty of the results. Fourth, the analysis allowed both short-term and long-term outcomes to be considered, including skeletal-related events, quality of life, and functional status; hence, the patient-centered impact of the interventions assessed was comprehensively evaluated. Finally, countries worldwide with different health systems were represented in the studies, increasing the generalizability of the findings across diverse patient groups.

#### 4.3.2 Limitations

Despite its advantages, several drawbacks must be acknowledged. To begin with, differences in study populations, disease severity, and intervention protocols might have affected comparability of outcomes, although, in general, statistical heterogeneity was low. Furthermore, the different types of pain-measurement tools employed in the studies, as well as the varying follow-up durations, might have introduced measurement bias and consequently affected the accuracy of the pooled estimates. In addition, some therapies, such as drug combinations, were assessed in fewer studies, which greatly limited the strength of the conclusions drawn for these subgroups. Moreover, the exclusion of non-English publications and unpublished data might have introduced publication bias, leading to an overestimation of treatment effects. The synthesis focused primarily on randomized controlled trials, which were considered over observational studies; thus, there might be a limitation on the extent of the insights about the real-world effectiveness and safety. Lastly, even though the meta-analysis was based upon pain severity and skeletal-related outcomes, other areas of patient experience, like psychosocial well-being and economic impact, were less frequently reported and hence could not be fully analyzed. The potential influence of age and gender on pain-management outcomes and highlighting the need for future studies to report stratified data.

## 5 Conclusion

The meta-analysis, including randomized controlled trials and comparative studies, together shows that radiotherapy, BMA, and radiopharmaceuticals can all be used to achieve pain relief in malignant bone tumors. One radiotherapy treatment session is just as effective as multiple sessions for pain relief; besides, it is more convenient, shorter, and less toxic. The BMAs consist of denosumab, zoledronic acid, ibandronate, and pamidronate, which always result in fewer skeletal-related events and better pain relief. Radiopharmaceuticals are good for pain relief and postponing skeletal complications, whether given alone or combined with chemotherapy or BMAs. There was a clear and consistent outcome across interventions, indicating that oncologists have multiple ways to relieve pain, preserve the skeleton, and even improve quality of life through safe and effective strategies for bone metastases from cancer.

## Data Availability

The raw data supporting the conclusions of this article will be made available by the authors, without undue reservation.
