# Correction: Global burden of pain in malignant bone tumors: a meta-analysis of severity and health outcomes

**DOI:** 10.3389/fonc.2026.1985547

**Published:** 2026-09-17

**Authors:** Guangda Zheng, Linghan Meng, Dongtao Li, Lu Shang, Juanxia Ren, Yanju Bao

**Affiliations:** 1 Department of Oncology, Guang’anmen Hospital, China Academy of Chinese Medical Sciences, Beijing, China; 2 Liaoning University of Traditional Chinese Medicine, Shenyang, Liaoning, China

**Keywords:** agents that modify bone, bisphosphonates, controlling pain, denosumab, malignant bone tumors, radiation therapy, radiopharmaceuticals

An incorrect **Funding** statement was provided. The correct **Funding** statement reads:

"The author(s) declared that financial support was received for this work and/or its publication. This work was supported by the Clinical Research and Achievement Transformation Capacity Enhancement Project for High-Level TCM Hospitals (No. HLCMHPP2023078), the Innovation Key Fund of the China Academy of Chinese Medical Sciences (No. 46117701), the Horizontal Scientific Research Project funded by Beijing Guang'an Institute of Traditional Chinese Medicine Oncology (Contract No. YY3101202507001), and the 6th Batch of National Excellent Talents in TCM Clinical Practice Training Program (Nos. 82778212 and 8277821)."

The original version of this article has been updated.

